# Radiometric and Morphologic Analysis of Arnold Chiari Type-I Malformation and Idiopathic Syringomyelia: A Case Series from Pakistan

**DOI:** 10.12669/pjms.40.12(PINS).11095

**Published:** 2024-12

**Authors:** Muhammad Tahir Khan, Muhammad Kaleem, Haseeb Mehmood Qadri, Hamza Manzoor, Mahwish Shoaib

**Affiliations:** 1 Dr. Muhammad Tahir Khan, FCPS Radiology. Post Graduate Resident, Department of Diagnostic Radiology, Punjab Institute of Neurosciences, Lahore, Pakistan; 2 Dr. Muhammad Kaleem, MD Radiology. Post Graduate Resident, Department of Diagnostic Radiology, Lahore General Hospital, Lahore, Pakistan; 3 Dr. Haseeb Mehmood Qadri, FCPS Neurosurgery. Post Graduate Resident, Department of Neurosurgery, Unit-I, Punjab Institute of Neurosciences, Lahore, Pakistan; 4 Dr. Hamza Manzoor, FCPS Radiology. Post Graduate Resident, Department of Diagnostic Radiology, Chughtai Health Care, Lahore, Pakistan; 5 Dr Mahwish Shoaib, FCPS Radiology. Assistant Professor, Department of Diagnostic Radiology, Punjab Institute of Neurosciences, Lahore, Pakistan

**Keywords:** Syringomyelia/diagnostic imaging, Chiari Malformation Type-I with Syringomyelia, Syringomyelia isolated, Magnetic Resonance Imaging, Hydrocephalus

## Abstract

**Background and Objective:**

Chiari I Malformation-associated syringomyelia (CM) and idiopathic syringomyelia (IS) are often confused together. They require different diagnostic approach and treatment modalities; it is important to distinguish between the two. We aimed to evaluate the radiological and morphologic characteristics of CM and IS in adult and pediatric patients in Pakistani population.

**Methods:**

Our retrospective case series was conducted at the Department of Radiology, Punjab Institute of Neurosciences assessing the operated cases of CM and IS cases from January 2022 to December 2023. Preoperative morphologic and radiological data was collected for adult and pediatric patients.

**Results:**

Out of 16 patients, 75% were adults and four were children. Twelve patients were male. The mean age of adult patients at presentation was 30.50 ± 9.20 years. There were nine cases of Chiari I Malformation and seven cases of idiopathic syringomyelia. Craniocervical junction (CCJ) was the most common site of syrinx formation in adults (41.66%) in both Chiari I and IS groups. The mean size of syrinx in adults in IS was 7.842 ± 1.93 and in CM was 4.740 ± 0.45. Mean size of syrinx in children was 4.84 ± 0.48 mm. Moniliform shape was the most common form of syrinx in patients in both CM and IS groups.

**Conclusion:**

In the Pakistani population, this study focuses on the radiological characteristics of idiopathic syringomyelia (IS) and Chiari I Malformation (CM), both of which commonly exhibit a moniliform syrinx. The craniocervical junction was frequently involved in CM, but IS exhibited a wider, bigger syrinx. Accurate diagnosis as well as treatment depend on imaging.

## INTRODUCTION

Chiari I Malformation (CM) is a group of neurological abnormalities that range from complete loss of the cerebellum to herniation of the cerebellar tonsils into the spinal canal. Hans Chiari initially described CM in 1890. Although the exact frequency of CM is unknown, Chiari Malformation Variety I is acknowledged as the most prevalent type, occurring in about 1 in 1,000 newborns and slightly more frequently in females (ratio of 1.3:1).^1^ Three to eight cases of CM are reported for every 100,000 people, and 65% of CM patients have been diagnosed with syringomyelia.^2^ Young adults (18–55 years old) are the target population for syringomyelia, which primarily affects levels of the thoracic spine and can occasionally result in holocord syrinx. Symptoms include paraparesis, hypoesthesia, ataxic gait, dysmetria, monoparesis, paresthesia, voiding dysfunction, and upper extremity atrophy, with notable muscle weakness and reduced sensation.^3^

Because CM and idiopathic syringomyelia (IS) require different treatment modalities, it is important to distinguish between the two. Both primary and secondary conditions, such as CM, can cause syringomyelia. When secondary, the goal of treatment is to address the underlying cause by, for example, releasing pressure that CM has placed on the brainstem and spinal cord. This distinction is crucial because CM entails the displacement of the cerebellum at the base of the skull, which obstructs the flow of spinal fluid and causes a fluid-filled cavity called a syrinx to form. This cavity can compress the spinal cord tissue and cause neurological symptoms.^2^,^3^

Notwithstanding the clinical importance of both disorders, there has been no research done in Pakistan on the radiological characteristics of CM and IS. Prior studies conducted in Pakistan have mostly focused on surgical results.^4^ By examining the radiological features of IS and CM, our study seeks to close this knowledge gap, presenting a fresh viewpoint in the regional medical literature and laying the groundwork for more intelligent diagnosis and treatment plans.

## METHODS

This retrospective, case series study was conducted in February and March 2024 at the Department of Radiology, Punjab Institute of Neurosciences, Lahore, Pakistan. Cases of CM and IS operated in the years 2022 and 2023 were included in this study. Complete preoperative records were retrieved from picture archiving and communication system (PACS). Conservatively managed cases were excluded from this study. Non-probability, consecutive inclusion technique was employed. Demographic data i.e. age, gender, radiometric data, i.e. size of the syrinx, anteroposterior and transverse diameters of posterior fossa and morphologic data, i.e. tonsillar descent, syrinx formation, level and shape of syrinx and associated craniocervical features were collected onto the Google Forms (Google Inc., USA). Measures of central tendency, frequency and percentages were calculated using Microsoft Excel Sheet (Microsoft Corporation, Washington, United States).

### Consent permission:

This was a retrospective study and presented no harm to the subjects studied. Exemption letter from Institutional Review Board was obtained with reference number- 1780/IRB/PINS/APPROVAL/2024.

### OPERATIONAL DEFINITIONS:

### Tonsillar herniation:

**Fig.1:**
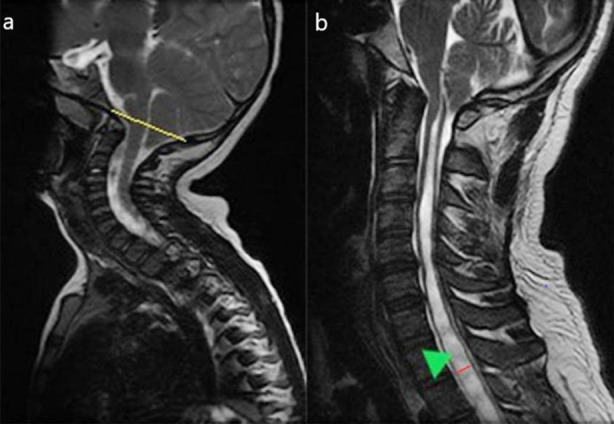
(a) Sagittal T2W sequence of MRI, showing tonsillar descent below the line drawn from basion to ophisthion (yellow line). (b) Sagittal T2W sequence of MRI, showing syrinx formation extending from craniocervical junction to dorsal spine level (green arrowhead). Red line depicting diameter of syrinx in anteroposterior dimension.

The descent of cerebellar tonsils through foramen magnum >5 mm below McRae line (line connects the anterior and posterior margins of foramen magnum i.e. basion to ophisthion) (Fig.1a).

### Syrinx:

A CSF filled cyst in the spinal cord, its diameter measured from anterior to posterior on sagittal or axial images (Fig.1b).

### Posterior fossa antero-posterior diameter:

Measurement of the posterior cranial fossa from the front to the back in an anteroposterior direction on sagittal or axial images. This line runs parallel to the base of the skull. Posterior fossa will be called small if diameter ≤ 60 mm.

### Posterior fossa transverse diameter:

Measurement of the posterior cranial fossa from the right to left in a transverse direction on coronal or axial images. Posterior fossa will be called small if diameter ≤ 99 mm. If both the AP and transverse diameters of posterior fossa are less than cut off values, then it will be labelled as small posterior fossa.

### Clival concavity:

**Fig.2:**
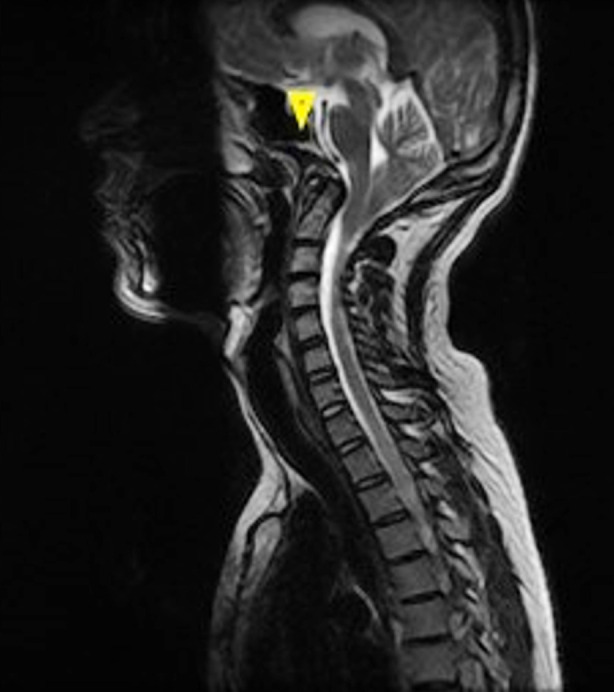
Sagittal T2W sequence of MRI showing clival concavity (yellow arrowhead).

Inward bending of the clivus (Fig.2).

## RESULTS

Out of 16 patients, 75% (12) were adults and four were children. The mean age of adult patients at presentation was 30.50 ± 9.20 years and that of children was 10.25 ± 4.78 years, respectively. Twelve patients (75%) were male and 25% were female. There were nine cases of Chiari I Malformation and seven cases of idiopathic syringomyelia, the former was more common in adults and both were equivocal in pediatric patients.

Among adults, craniocervical junction was the most common site of syrinx formation in 41.66% cases and the caudal extent of syrinx varied in these five cases. The mean size of syrinx was 6.79 ± 2.12 mm for nine adult patients. The mean anteroposterior diameter of posterior fossa for adults was 70.13 ± 13.53 mm, while the mean transverse diameter of posterior fossa was 86.99 ± 9.32 mm. Hydrocephalus and foramen magnum obstruction were present in 16.67% and 33.33% patients, respectively and only involving patients with CM (Table-I).

**Table-I:** Summary and radiometric analysis of adult patients with Chiari I Malformation (CM) and Idiopathic Syringomyelia (IS).

Pt	Age (y)	Gender	Dx (CM/IS)	Syrinx level	Tonsillar descent	Syrinx diameter (mm)	PF diameter (mm)	HCP (+/-)	FMO (+/-)
**1**	25	M	CM	CCJ-D4	+	7.29	68 AP, 112 T	+	+
**2**	42	M	CM	C2-D9	+	6.1	61 AP, 100 T	-	-
**3**	17	M	CM	No	+	Nil	71 AP, 101 T	-	-
**4**	20	M	CM	C2-D7	+	5.57	67 AP, 113 T	-	+
**5**	31	F	CM	No	+	Nil	68 AP, 106 T	+	+
**6**	25	M	CM	C7-D3	+	3	62 AP, 104 T	-	-
**7**	38	M	CM	No	+	Nil	66 AP, 105 T	-	+
**8**	39	M	IS	CCJ-D4	+	5.97	110 AP, 78 T	-	-
**9**	29	F	IS	C2-D5	-	6.85	69 AP, 111 T	-	-
**10**	29	F	IS	CCJ-D5	-	9.71	56 AP, 111 T	-	-
**11**	47	M	IS	CCJ-D7	-	10	76 AP, 107 T	-	-
**12**	24	M	IS	CCJ-D11	-	6.68	68 AP, 105 T	-	-

Legend: Pt: patient, M: male, F: female, CCJ: craniocervical junction, present: +, absent -, PF: posterior fossa, AP: anteroposterior, T: transverse, HCP: hydrocephalus, FMO: foramen magnum obstruction.

The mean syrinx diameter in our adult patient study showed a significant difference between individuals with idiopathic syringomyelia (IS) and patients with Chiari I malformation (CM). The average syrinx diameter in CM patients was found to be 4.740 mm, but it was significantly greater in IS patients, measuring 7.842 mm on average. According to this analysis, syrinx linked to Chiari I malformation are often much narrower than those linked to idiopathic syringomyelia. This finding may have consequences for how these conditions are treated and presented clinically (Table-I).

Among pediatric patients, syrinx was present in two patients, involving lumbar and cervical spinal cord. The mean size of syrinx was 4.84 ± 0.48 mm for two patients. The mean anteroposterior diameter of posterior fossa for adults was 69.00 ± 8.08 mm, while the mean transverse diameter of posterior fossa was 94.75 ± 16.09 mm. Hydrocephalus and foramen magnum obstruction were present in 25% and 50% patients, respectively (Table-II).

**Table-II:** Summary and radiometric analysis of pediatric patients with Chiari I Malformation (CM) and Idiopathic Syringomyelia (IS).

Pt	Age(y)	Gender	Dx (CM/IS)	Syrinx level	Tonsillar descent	Syrinx diameter (mm)	PF diameter (mm)	HCP (+/-)	FMO (+/-)
13	4	M	CM	No	+	Nil	64 AP, 78 T	-	-
14	14	M	CM	No	+	Nil	62 AP, 103 T	+	+
15	9	M	IS	C2-C3	+	5.18	80 AP, 113 T	-	+
16	14	F	IS	L1-L2	-	4.5	70 AP, 85 T	-	-

Legend: Pt: patient, M: male, F: female, CCJ: craniocervical junction, present: +, absent -, PF: posterior fossa, AP: anteroposterior, T: transverse, HCP: hydrocephalus, FMO: foramen magnum obstruction.

**Fig.3:**
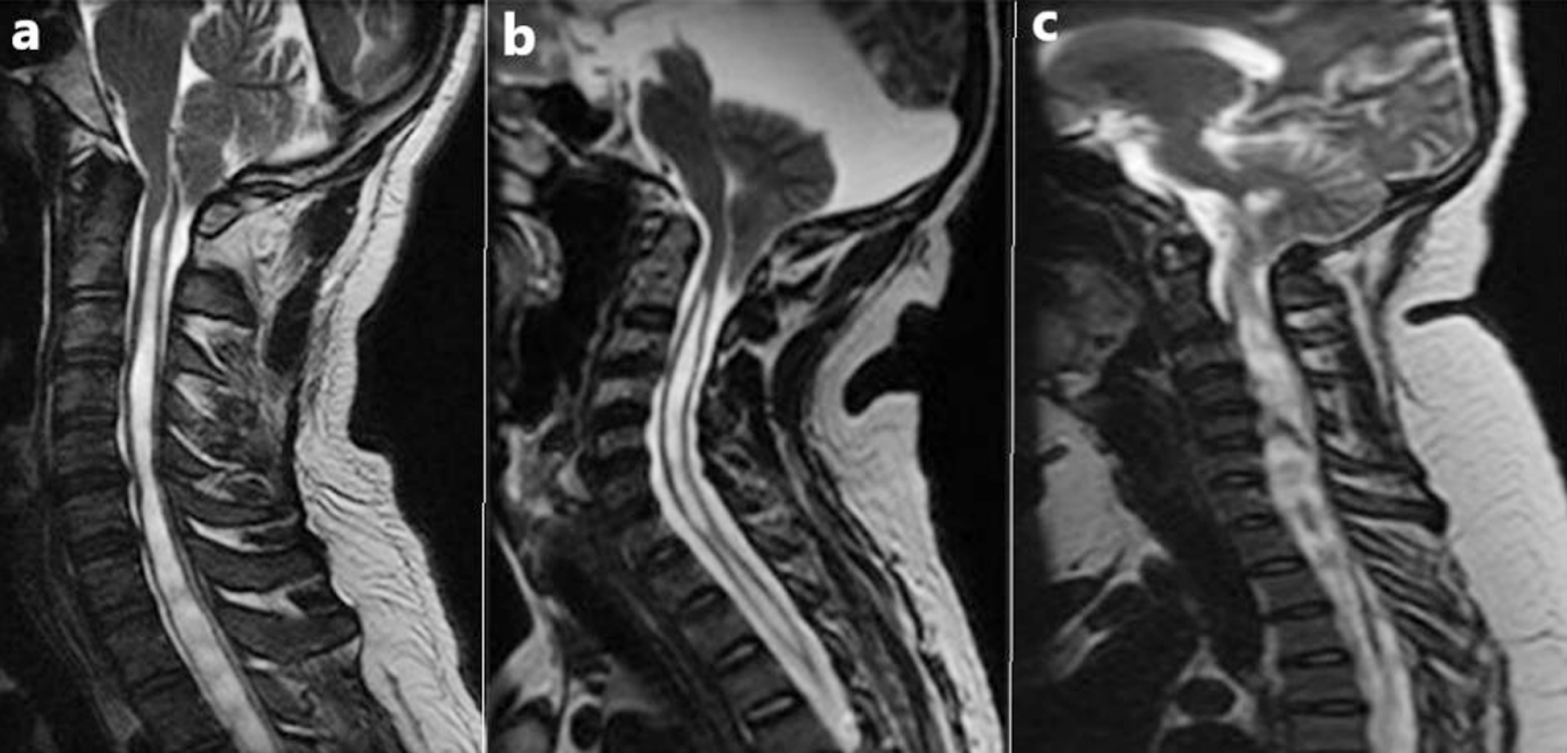
Types of syrinx. (a) Moniliform, (b) Slender and (c) Circumscribed type.

Overall, the most common morphology of syrinx was moniliform type, seen in 54.54% patients, while both pediatric patients with syrinx had slender type (Table-III and Fig.3). In our study, patients with idiopathic syringomyelia (IS) and those with Chiari I malformation (CM I) had different syrinx shapes. Of the four CM I patients, two had a syrinx that was primarily moniliform in shape, one had a syrinx that was circumscribed, and one patient had a slender syrinx. In contrast, the syrinx was most frequently moniliform in four of the seven individuals in the idiopathic syringomyelia group, whereas the remaining three patients had a slender syrinx. These results states that moniliform syrinx was common in both CM and IS patients (Table-III).

**Table-III:** Morphologic features of adults and children with Chiari I Malformation (CM) and Idiopathic Syringomyelia (IS).

Pt	Ventral CSF (I/D)	Dorsal CSF (I/D)	Platybasia (+/-)	Clival Concavity (+/-)	Shape of Syrinx
1	D	D	-	-	Moniliform
2	I	I	-	-	Moniliform
3	I	D	-	-	Nil
4	I	D	-	+	Moniliform
5	I	D	-	+	Nil
6	D	I	-	-	Slender
7	D	I	+	-	Nil
8	D	I	-	-	Moniliform
9	I	D	-	-	Circumscribed
10	I	D	-	-	Moniliform
11	D	D	-	+	Slender
12	D	I	-	-	Moniliform
13	I	I	-	-	Nil
14	D	I	-	-	Nil
15	I	I	-	-	Slender
16	I	D	-	-	Slender

Legend: I: increased, D: decreased, present: +, absent -.

## DISCUSSION

Punjab Institute of Neurosciences (PINS) is the largest neuro-spine, public sector facility in Pakistan. Only 16 cases out of 6000 elective surgeries performed in 2022 and 2023 at our institution were found to have IS and CM, accounting to 0.27% of the total operated cases. The stunningly low frequency of these conditions at our center is consistent with the existing scientific literature. Kahn et al. have analyzed the image prevalence of CM in various studies in the range of 0.24%-3.4% in general population.^5^ Another reason for the low reporting of this condition at our institution is the exclusion of cases that were conservatively managed. No publication on radiological and morphological features of CM and IS in Pakistani population was found.

A Russian study by Bogdanov et al. showed that the mean age of patients with CM and IS was 48.7 ± 11.6 and 49.4 ± 10.9 years, respectively. Males made up 13 out of 17 patients in the CM group and 15 out of 17 patients in IS group, suggesting a higher male predominance.^6^ This gender-based trend is consistent with the findings of our study with majority male patients. However, retrospective study documented by Chinese doctors in 2019 highlighted opposite trends; a female majority of 58.3% in both groups of CM and IS.^7^ Precise comparisons on gender predilection are not possible due to scarcity of existing literature. Nonetheless, physiological and genetic factors in males could be held accountable.

Tonsillar descent was noted in all cases of adult CM in our study, but two of them had hydrocephalus and foramen magnum occlusion concomitantly. On the other hand, another two patients had foramen magnum obstruction without hydrocephalus, indicating complex morpho-pathologic aspects of CM. Occurrence or absence of hydrocephalus is no longer considered a diagnostic criterion of CM and IS, but it has been reported in association with both conditions off and on.^3^

The results show that the posterior fossa dimensions were within the normal range in all cases. This is contradictory to the widely held belief that cerebellar maldevelopment, rather than the posterior fossa’s incapacity to house its neural contents owing to insufficient internal volume, is typically the cause of CM.^1^ Fifty percent individuals with Chiari I Malformation (CM) had syrinx formation in the cervico-dorsal spinal cord. Zhu et al. studied the characteristic of scoliotic deformity in patients with CM versus IS. Two locations were equivocally found, 45.9% each, as the most common site of syrinx formation, i.e. cervical and cervico-thoracic spinal cord.^8^ The proximal location of syrinx in spinal cord could be well attributed to the localized effects of tonsillar descent across foramen magnum with each heartbeat in a pulsatile manner.^7^

CM-associated syrinx and IS are two different entities. The former is thought to be associated with factors, like older age, pegged shaped of tonsils and basilar invagination, while atlantoaxial instability, narrowing of cervical spinal canal diameter and decreased compliance of draining veins of spinal cord are thought to contribute to the development of latter.^5^,^7^

We looked into idiopathic syringomyelia (IS) in adults and the craniocervical junction was shown to be the most common site for syrinx development in 41.66% of cases. This is comparable with a study conducted by Tan and colleagues in 2018, showing that 43.6% patients had their syrinx located in the cervical spine, while 41.8% had it in the thoracic region.^9^ This heterogeneity in the position of the syrinx highlights the distinctive features of IS apart from CM-associated syringomyelia and the varied anatomical appearance of the condition. On the other hand, Bogdanov et al. described overlapping clinical and neuroimaging characteristics, such as a smaller posterior fossa and signs of central cervical myelopathy, indicating a shared developmental mechanism for these disorders.^6^ But our results highlight the necessity for a distinctive diagnostic approach for adult and pediatric CM and IS.

None of our pediatric cases with CM had syrinx, while one of the two patients with IS had syrinx in the lumbar spinal cord. There is scarcity of literature on pediatric idiopathic syringomyelia. Magge et al. conducted a multi-centric study in the United States including 48 children with the diagnosis of idiopathic syringomyelia. Thoracic spinal cord was found as the most common site of syrinx formation in 52% patients.^10^ Seventy-five percent pediatric patients at had increased levels of CSF in the ventral and dorsal subarachnoid spaces and showed a more uniform trend than the adult patients. Adult patients at our center had highly variable levels of CSF in these spaces. We ordered plain MRI for all our patients. In contrast, Hwee et al. advocate the utilization of cine phase-contrast MRI in CM with syringomyelia especially. Cine phase-contrast MRI is a known diagnostic approach to detect the degree of CSF outflow obstruction. It also helps in surgical selection of patients and gauge preoperative and postoperative difference in symptomatology.^11^

Lu et al. have studied the relationship of moniliform-type syrinx and clinical outcomes of operated cases in 2022. Patients with moniliform-type syrinx have short preoperative duration of clinical manifestations and better resolution of syrinx than those with non-moniliform type syrinx.^12^ We also found this type of syrinx as the most common one in our patients. This is in contrast to the findings of other group of Chinese researchers who found circumscribed-type as the most common syrinx morphology in 65.57% patients. Regional and genetic differences in syrinx shape might account for these differences.^8^

### Limitations:

The small sample size, retrospective nature and mono-centricity of this study limit the generalizability of the findings across broader populations and varied clinical settings.

## CONCLUSIONS

This study offers significantly insights into the demographic and radiological spectrum of Chiari I Malformation and idiopathic syringomyelia in Pakistani population. Moniliform syrinx morphology was seen most frequently in both groups. The most common site of occurrence of syrinx was craniocervical junction. In most cases, CM was linked to a moniliform syrinx at the craniocervical junction, which occasionally extended into the cervical spine. In other cases, hydrocephalus and foramen magnum obstruction were also present. In contrast, IS was characterized by a bigger syrinx, which was most frequently more widely distributed along the spinal cord in a moniliform or slender morphology. These results underscore the importance of imaging in the diagnosis and treatment of both conditions and the complexity of syringomyelia in both.

### Authors Contribution:

**MTK** collected data and contributed to manuscript writing and responsible for the accuracy the study.

**MK** collected data and contributed to manuscript writing.

**HMQ** conceived, designed the study, analyzed data and contributed to manuscript writing and critical review.

**HM** collected data and contributed to manuscript writing.

**MS** supervised the study and critically reviewed the manuscript.

All authors have read and approved the final version of the manuscript.
